# Estimation of 3D Ground Reaction Force and 2D Center of Pressure Using Deep Learning and Load Cells Across Various Gait Conditions

**DOI:** 10.3390/s25113357

**Published:** 2025-05-26

**Authors:** Junggil Kim, Ki-Cheon Kim, Gyerae Tack, Jin-Seung Choi

**Affiliations:** 1 Department of Biomedical Engineering, Konkuk University, Chungju 27478, Republic of Korea; ggkk007@kku.ac.kr (J.K.); tigertwin@kku.ac.kr (K.-C.K.); grtack@kku.ac.kr (G.T.); 2 BK21 Plus Research Institute of Biomedical Engineering, Konkuk University, Chungju 27478, Republic of Korea

**Keywords:** ground reaction force, center of pressure, deep learning, estimation, load cell, gait

## Abstract

Traditional force plate-based systems offer high measurement precision but are limited to laboratory settings, restricting their use in real-world environments. To address this, we propose a method for estimating a three-axis ground reaction force (GRF) and two-axis center of pressure (CoP) using a shoe embedded with three uniaxial load cells. The estimation was conducted under five gait conditions: straight walking, turning, uphill, downhill, and running. Data were collected from 40 healthy young adults. Four deep-learning models—Fully Connected Neural Network (FCNN), Convolutional Neural Network (CNN), Sequence-to-Sequence Long Short-Term Memory (Seq2Seq-LSTM), and Transformer—were evaluated. Among them, Seq2Seq-LSTM and CNN achieved the highest performance in predicting both GRF and CoP. However, the medio-lateral (ML) components showed lower accuracy than the vertical and anterior–posterior directions. In slope conditions, particularly for vertical GRF, relatively higher root mean-square error (RMSE) values were observed. Despite some variation across gait types, predicted values showed high agreement with measurements. Compared with previous studies, the proposed method achieved comparable or better performance with a minimal sensor setup. These findings highlight the feasibility of accurate GRF and CoP estimation in diverse gait scenarios and support the potential for real-world applications. Future work will focus on sensor optimization and broader population validation.

## 1. Introduction

Gait is one of the most fundamental human movements, driven by the propulsion force generated through foot movement and ground contact [1]. During walking, the body requires complex coordination between the nervous and musculoskeletal systems to maintain stability and generate forward momentum [2]. Patients with dysfunction in either system often face challenges in gait execution, resulting in gait impairments.

Quantitative gait metrics are essential for objective assessment and play a critical role in the clinical diagnosis and treatment evaluation of patients with gait disorders. These metrics contribute to improved prognosis and prevention of disease progression. Among the most widely used indicators are ground reaction force (GRF) and center of pressure (CoP), which are utilized extensively in both clinical practice and academic research. GRF and CoP have been applied in studies analyzing gait patterns in patients with spinal cord injury, Parkinson’s disease, cerebral palsy, and multiple sclerosis, as well as in healthy control groups [3,4,5]. They are also used to assess balance in older adults, aiding in the prediction of fall risk and cognitive decline [6,7].

GRF and CoP are essential for evaluating biomechanical aspects of gait, such as weight transfer, shock absorption, braking forces, and balance stability [8,9,10]. GRF includes three-axis (X, Y, Z) components, and CoP represents the spatial distribution of forces, making it important to integrate information across all axes for comprehensive analysis [11,12].

Traditionally, GRF and CoP are measured using force plates, which are considered the gold standard due to their high precision. However, force plates are limited to laboratory settings and require additional setups to capture multiple steps during walking. Furthermore, gait experiments conducted in lab environments may not reflect real-world conditions due to psychological influences on participants, controlled floor surfaces, and variations in footwear usage [13,14,15,16]. Therefore, there is a growing need for methods that can measure GRF and CoP under more realistic conditions.

As a promising alternative, in-shoe pressure sensors have been used to estimate GRF and CoP. In our previous study, we developed a shoe embedded with three uniaxial load cells and employed a Sequence-to-Sequence Long Short-Term Memory (Seq2Seq-LSTM) model to estimate three-axis GRF. In that study, GRF estimation during straight walking achieved root mean-square error (RMSE) values of 65.12 N (vertical), 15.50 N (anterior–posterior), and 9.83 N (medio-lateral), with corresponding correlation coefficients of 0.97, 0.96, and 0.90, respectively. These results were comparable to or exceeded those reported in previous studies [17].

Numerous studies have utilized force-sensing resistors (FSRs) to estimate GRF and CoP. For example, a study using 99 FSRs reported RMSE values of 5 N in the anterior–posterior direction and 21 N in the vertical direction, with correlation coefficients of 0.91 and 0.83, respectively [18]. Another study using six FSRs reported a CoP RMSE of 9.17 mm with a correlation of 0.98, and a vertical GRF RMSE of 34.01 N with a correlation of 0.95 [19]. A separate study using 16 FSRs to estimate vertical GRF under various running intensities achieved a correlation coefficient of 0.999, suggesting that even high-intensity GRF can be accurately estimated using pressure sensors [20]. Beyond straight walking, GRF estimation has been explored under various gait conditions using different methodological approaches. For turning walking, Eguchi et al. (2023) utilized a single-axis load cell with a maximum likelihood model [21], whereas Yamaguchi et al. (2023) applied Gaussian process regression with a three-axis load cell [22]. In slope walking, Honert et al. (2022) estimated three-axis GRF using a single-axis load cell [18].

Since gait strategies and the resulting GRF and CoP patterns vary significantly depending on the walking condition, such as slope walking, turning, and running, it is important to collect data across multiple gait types and develop prediction models accordingly [23,24,25]. However, most previous studies have analyzed each gait in isolation, often focusing on a single type of locomotion per study. This condition-specific approach limits the generalizability of the models and highlights the need for integrated analyses across diverse walking conditions.

In this study, we aim to estimate 3D GRF and 2D CoP across various gait conditions—straight, turning, uphill, downhill, and running—as an extension of our previous work. In addition to the Seq2Seq-LSTM model, we evaluate and compare several deep-learning architectures, including Fully Connected Neural Networks (FCNN), Convolutional Neural Networks (CNN), and transformer models, to identify the most suitable approach for each gait type. Each model offers a unique perspective on how GRF and CoP can be predicted based on its training mechanism. The FCNN model enables investigation into whether GRF and CoP can be predicted through feedforward mappings of load-cell signals without explicit temporal modeling. The CNN model, by applying convolutional filters, captures local temporal patterns, allowing for the assessment of whether short-range dynamics in the input sequence can support accurate force and pressure prediction. The transformer model, leveraging the attention mechanism, enables learning of long-range dependencies across the entire sequence, thereby allowing the estimation of GRF and CoP based on global temporal relationships. By evaluating multiple learning models across diverse gait conditions, this study aims to identify condition-specific optimal models and provide insight into the relationship between gait dynamics and model architecture characteristics.

## 2. Materials and Methods

### 2.1. Experiment

This study was conducted with 40 healthy young adults in their 20s. Demographic and physical characteristics, including age, height, weight, dominant foot, and foot size, were recorded for each participant. All participants were thoroughly informed about the purpose and procedures of the study, and written consent was obtained prior to participation. To ensure transparency in the data-acquisition process, no anticipated outcomes, such as potential differences in results across gait conditions, were disclosed to the participants. The study protocol was approved by the Institutional Review Board of Konkuk University (Approval No. 7001355-202307-HR-676). A summary of participant characteristics is provided in Table 1.

### 2.2. Shoe with Three Uniaxial Load Cells

**Figure 1:**
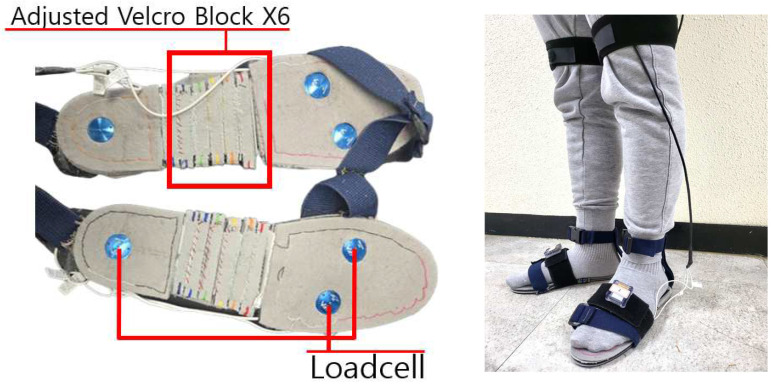
Custom shoe embedded with three uniaxial load cells. The detailed rated capacities of the sensors used are shown in Table S1. A detachable central block with a width of 10 mm is located in the middle of the shoe, allowing the shoe size to be adjusted in 10 mm increments. This enables precise positioning of the load cells at the heel and at the first and fifth metatarsal heads according to each participant’s foot size. Data collection and storage were performed using Python 3.7 at a sampling frequency of 30 Hz.

In this study, we used a custom-made shoe embedded with three uniaxial load cells (EzForce-1D, i2A Systems Inc., Daejeon, Republic of Korea). The design of the shoe and its theoretical framework were based on our previous work [17], and the shoe is illustrated in Figure 1.

### 2.3. Data Collection and Processing

**Figure 2:**
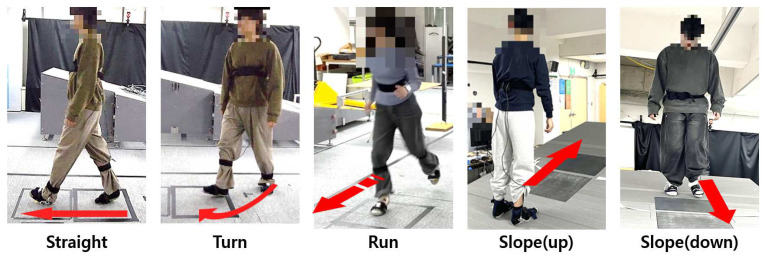
Five gait conditions evaluated in the experiment. Participants wore the custom shoe embedded with load cells and performed all five gait types at their preferred walking speed.

In this study, five gait conditions were evaluated: straight walking, turning, uphill, downhill, and running. Figure 2 illustrates the examples of the gait experiments. Each participant performed five-to-seven trials for each condition. All participants were instructed to walk at their self-selected speed for each gait condition. For the straight, turn, and run conditions, two force plates (AMTI, Watertown, MA, USA) embedded in a level walkway were used. For the slope (uphill/downhill) condition, participants walked on a 13° inclined ramp equipped with an embedded force plate (Kistler instrumented AG, Winterthur, Switzerland). GRF and CoP data were collected at a sampling frequency of 120 Hz using Cortex 4.0 software (Motion Analysis Corp., Rohnert Park, CA, USA).

Although load cell data were continuously collected throughout the entire walking trial, only the segments corresponding to contact with the force plate were selected for analysis. To reduce signal noise, a fourth-order Butterworth low-pass filter with a cutoff frequency of 10 Hz was applied. Load-cell data were synchronized with GRF and CoP measurements at the moment of heel strike, which marked the onset of data recording for both systems. Force-plate data were always taken at 0 N, making it simple to determine the heel strike. Since the load-cell sensor always makes contact with the foot, even during the swing phase, very small values were recorded for the load-cell data. When resampling and filtering were conducted, these noises were converted to close to zero, and the identical heel strike as the force plate could be determined. In the CoP signals, ground artifacts occurring at heel strike and toe-off were observed and removed by applying a threshold based on the vertical GRF signal [26,27]. For gait conditions where an appropriate threshold has not been clearly established, such as turning, running, and slope walking, statistical analyses were conducted based on the peak vertical GRF values. In conditions showing statistically significant differences, specifically running and downhill walking, thresholds were determined using a ratio of the maximum vertical GRF, as detailed in the Table 2. The filtered signals were resampled to 100 data points using spline interpolation. This allowed for the normalization of the stance phase to a percentage scale, facilitating direct comparison across trials and participants.

### 2.4. Model

**Figure 3:**
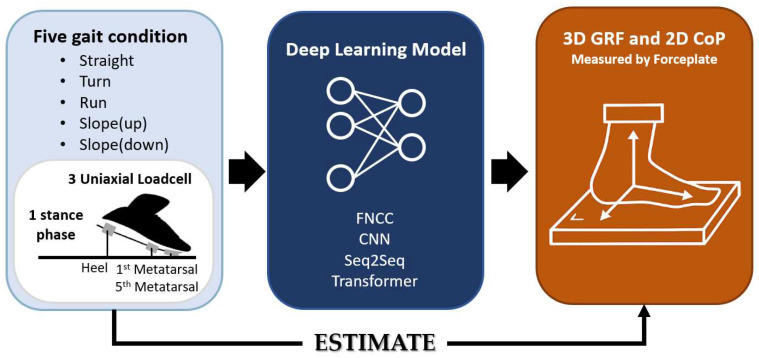
Overview of the estimation model for 3D GRF and 2D CoP. The input data consist of filtered signals from load cells located at the heel, first metatarsal, and fifth metatarsal. The output data represent filtered ground truth GRF and CoP measurements obtained from the force plate.

In this study, four deep-learning models were used to estimate GRF and CoP: Fully Connected Neural Network (FCNN), Convolutional Neural Network (CNN), Sequence-to-Sequence Long Short-Term Memory (Seq2Seq-LSTM), and transformer. The input data consisted of signals from three uniaxial load cells embedded in the shoe, while the output data included GRF components (vertical, anterior–posterior, and medio-lateral) and CoP components (anterior–posterior and medio–lateral) measured by the force plate. Hyperparameters for each model were optimized within the value ranges presented in Supplementary Table S2. Model performance was evaluated based on root mean-square error (RMSE) and Pearson correlation coefficients [19,20,28]. Figure 3 presents the full pipeline of the model training process.

### 2.5. Validation

To evaluate the generalizability of the models, 60% of participants were allocated to the training set, 20% to the validation set, and the remaining 20% to the test set. To ensure dataset independence, data from the same participant were not shared across different sets. Model performance was evaluated using root mean-square error (RMSE) and Pearson correlation coefficients. Additionally, Bland–Altman analysis was conducted to assess the agreement between the predicted and measured GRF and CoP values. The following four metrics were derived from the Bland–Altman analysis:

- Bias: the mean difference between predicted and measured values
- 95% confidence interval (CI) of the bias, indicating the magnitude of systematic error
- Limits of agreement (LoA): the 95% CI of the difference, representing the agreement range between two measurements
- 95% CI of the LoA: the precision or error bounds of the upper- and lower-LoA values

## 3. Results

Data were excluded from analysis if the participant’s foot partially missed the force plate or if data were not properly collected due to communication issues. The final number of valid trials per gait condition is summarized in Table 3.

**Figure 4:**
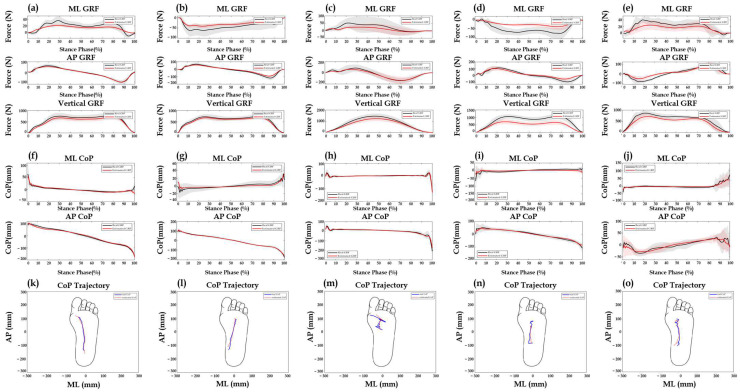
Average trajectories of GRF and CoP for the test set. Estimated GRF values are shown for each gait condition: (**a**) straight walking, (**b**) turning, (**c**) running, (**d**) uphill, and (**e**) downhill. Estimated CoP values are shown for: (**f**) straight walking, (**g**) turning, (**h**) running, (**i**) uphill, and (**j**) downhill. The experimental mean and standard deviation are represented by black solid lines and shaded areas, respectively. The estimated values are shown as red solid lines with red shaded areas. (**k**−**o**) illustrate representative CoP trajectories in the 2D plane for each corresponding gait condition.

**Figure 5:**
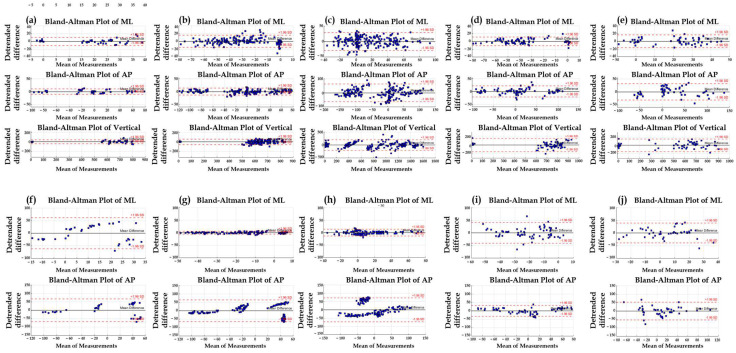
Bland–Altman plots for GRF and CoP across gait conditions. (**a**–**e**) show Bland–Altman results for GRF estimation under the following conditions: (**a**) straight walking, (**b**) turning, (**c**) running, (**d**) uphill, and (**e**) downhill. (**f**–**j**) show Bland–Altman results for CoP estimation under: (**f**) straight walking, (**g**) turning, (**h**) running, (**i**) uphill, and (**j**) downhill.

Table 4 presents the selected neural network model and the corresponding hyperparameters for each gait condition. The final model was selected according to the RMSE obtained on the validation set, as shown in Figure 4. For GRF estimation, the Seq2Seq-LSTM model was selected as the final model for straight walking, running, and uphill walking, whereas the CNN model was selected for turning and downhill walking conditions. For CoP estimation, the Seq2Seq-LSTM model was chosen for straight, turning, running, and uphill walking, while the CNN model was selected for downhill walking. Model-performance metrics for each gait condition are provided in Supplementary Tables S3 and S4. The results of the Bland–Altman analysis are shown in Figure 5 and Table 5. According to the Bland–Altman results, most data points lie within LoA, and the scatter plot exhibits a randomly dispersed pattern around the mean difference, indicating that the variability is evenly distributed without systematic bias.

## 4. Discussion

In this study, we estimated three-axis GRF and two-axis CoP across five gait conditions using a shoe embedded with three uniaxial load cells. Four deep-learning models, FCNN, CNN, Seq2Seq-LSTM, and transformer, were evaluated. To identify the optimal model for each condition, we trained the models by adjusting hidden layer configurations, parameters, and learning rates.

Among the models, CNN and Seq2Seq-LSTM consistently demonstrated superior performance. FCNN and transformer were not selected for any of the gait conditions. The limited performance of the FCNN model is likely due to its inability to capture temporal dynamics, as it processes input data frame-by-frame without considering time dependencies. Although the transformer model generally performs well with long time-series data, its effectiveness may diminish in relatively short gait sequences [29]. In contrast, CNN effectively captured local force patterns, while Seq2Seq-LSTM was able to model both short- and long-term temporal dependencies.

Overall, the estimation accuracy of ML GRF and CoP was lower than that of the other directions. This finding is consistent with previous studies [17] and may be attributed to the small magnitude of the ML signals, variability in individual gait styles, balance ability, and environmental factors [30]. For instance, a study by Oubre et al. (2021) reported low accuracy in ML GRF prediction, even when using six sensors placed at the heel, first and fifth metatarsal heads, lateral arch, and big toe. In that study, the correlation coefficient for ML GRF was only 0.51 [28], suggesting that ML forces are highly variable and difficult to predict precisely using a limited number of sensors.

The lowest ML GRF prediction accuracy was observed during running. Due to the higher velocity and shorter contact time with the ground in running, lateral balance control becomes more dynamic [31], increasing variability both across and within participants during repeated trials.

Low prediction accuracy for ML CoP was also observed during slope walking. This may be due to changes in foot progression angle required to generate greater propulsion and braking forces on an incline [9,24]. In particular, medial rotation of the foot can reduce lateral movement and energy expenditure [32,33]. In our study, CoP trajectories during slope walking showed considerable inter-subject variability, which likely contributed to reduced ML CoP prediction accuracy. Similarly, CoP prediction during running was less accurate, likely due to increased variability in AP and ML CoP components caused by the shorter ground contact time.

Vertical GRF estimation during uphill walking showed relatively high RMSE. This may be due to changes in foot–ground interaction that result in altered joint flexion angles and muscle activation patterns [34]. When ascending a slope, individuals tend to increase hip and knee flexion and engage stronger ankle dorsiflexion to lift the foot higher. Forward weight shift also increases instability [35], thereby increasing variability in vertical GRF. In a study by Lay (2005), 3000 repeated gait trials on slopes with inclines ranging from 0% to 39% showed increased standard deviation in average GRF [24]. Similarly, our study found that uphill GRF patterns varied across participants, which likely contributed to the lower estimation accuracy.

Although RMSE values were relatively high for some gait conditions, the overall GRF prediction accuracy was comparable to or higher than those reported in previous studies. For straight walking, our RMSE results were comparable to those reported by Oubre et al. (2021)—25.9, 10.7, and 72.8 N for ML, AP, and vertical direction, respectively—and were even lower in the ML and AP directions [28]. Furthermore, our correlation coefficients for GRF exceeded those of Oubre et al. across all directions.

In the study by Kammoun et al. (2024), GRF was estimated using 16 pressure sensors, and Seq2Seq-LSTM demonstrated the highest performance. Similar to our findings, the vertical GRF yielded the highest correlation coefficient. However, the reported RMSE was higher than in our study [36]. Honert et al. (2022) estimated GRF during slope walking using FSR array insoles and achieved correlation coefficients of 0.90 and 0.99 for AP and vertical GRF, respectively [18]. In comparison, our results were slightly lower (uphill: AP 0.89, vertical 0.93; downhill: AP 0.93, vertical 0.94). This may be due to the higher spatial resolution of the FSR sensors (1.5 cm^2^), which were distributed across the entire plantar surface in the Honert study.

For turning, Eguchi et al. (2023) used 15 FSRs and a maximum likelihood-based model to estimate GRF (%BW) during walking along a 1 m-radius circular path, reporting NRMSEs of 4.8, 5.2, and 4.1 for ML, AP, and vertical GRF, respectively [21]. In our study, GRF (%BW) was derived from absolute GRF (N), and the corresponding NRMSEs were 1.9, 1.4, and 5.4, indicating better performance in ML and AP directions.

The proposed load-cell-embedded shoe and the associated deep-learning models demonstrated comparable or superior estimation performance relative to previous studies. These results suggest that the system is sufficiently accurate for estimating GRF during overground walking outside controlled laboratory environments. However, several limitations should be noted.

First, the current sensor configuration was not sufficient to capture variability in the ML direction. Compared to studies utilizing high-density FSR arrays [18], future work should explore sensor number and placement optimization to improve ML estimation accuracy.

Second, participants walked at their self-selected speeds, and the number of training samples may have been insufficient to fully capture individual gait patterns. Collecting a more diverse dataset, including variations in anthropometrics and walking speeds, could enable the model to better learn inter-subject variability.

Third, the participants were limited to healthy young adults in their 20s. It remains unclear whether the trained models can generalize to other age groups with different physical characteristics. Moreover, the proposed approach is not yet suitable for populations with greater variability in gait, such as patients with stroke. Future research should aim to extend this work to clinical populations and explore model-adaptation strategies for pathological gait.

## 5. Conclusions

The load-cell-embedded shoe and deep-learning models proposed in this study demonstrated high accuracy in estimating 3D GRF and 2D CoP across various gait conditions. Among the models evaluated, Seq2Seq-LSTM and CNN consistently showed superior performance. However, estimation accuracy for the medio-lateral (ML) direction was relatively lower under certain gait conditions. This limitation is attributed to the current sensor configuration of the proposed shoe, suggesting the need for further research to address this issue.

## Figures and Tables

**Table 1:** Subjects’ characteristics.

Age	Height	Weight	Dominant Foot (R/L)	Foot Size	Gender (M/F)
24.56 ± 2.47	169.32 ± 8.83	71.64 ± 16.79	41/2	256.28 ± 19.03	29/14

**Table 2:** Maximum ground reaction force (GRF) values across gait conditions.

Trial	Straight	Turn	Run	Slope (Up)	Slope (Down)
Max Vertical GRF (N)	757.70 ± 198.15	719.18 ± 165.77	1417.57 ± 295.94	792.9 ± 204.45	878.95 ± 258.59
p (With Normal)	-	0.053	0 *	0.15	0 *
Threshold (N)	20	20	37.42	20	23.2

* Significant difference from the straight condition based on independent *t*-tests.

**Table 3:** Number of valid trials used for each analysis.

Trial	Straight	Turn	Run	Slope (Up)	Slope (Down)
n of data	126	228	196	151	118

**Table 4:** Final neural network model, hyperparameters, and performance metrics for each gait condition. The table summarizes the selected model architecture, optimized hyperparameters, and evaluation results, including Pearson correlation coefficient, root mean-square error (RMSE), and normalized RMSE (NRMSE), for each GRF and CoP estimation task across the five gait conditions. RMSE of the GRF is presented in Newton (N), RMSE of the CoP is presented in millimeters (mm), and NRMSE is presented as a percentage (%).

Trial	Straight		Turn		Run		Slope (up)		Slope (down)	
	GRF	CoP	GRF	CoP	GRF	CoP	GRF	CoP	GRF	CoP
Method	Seq2Seq	Seq2Seq	CNN	Seq2Seq	Seq2Seq	Seq2Seq	Seq2Seq	Seq2Seq	CNN	CNN
Layers	4	4	2	3	4	2	4	1	4	2
Initial Units	100	200	4	100	100	200	100	100	32	4
LR	0.01	0.001	0.001	0.01	0.01	0.01	0.01	0.01	0.001	0.1
ML Correlation	0.88	0.86	0.80	0.65	0.67	0.51	0.91	0.22	0.76	0.33
AP Correlation	0.99	0.99	0.98	0.99	0.90	0.79	0.93	0.93	0.89	0.76
Vertical Correlation	0.99	-	0.98	-	0.98	-	0.94	-	0.91	-
ML RMSE (NRMSE)	11.23 (22.57)	5.64 (15.18)	24.32 (30.32)	7.16 (15.00)	33.24 (37.67)	6.27 (14.05)	35.48 (33.99)	10.52 (13.71)	17.12 (29.32)	8.95 (24.31)
AP RMSE (NRMSE)	11.55 (6.73)	10.12 (4.00)	18.10 (9.60)	8.48 (3.57)	48.90 (19.29)	10.04 (10.53)	36.44 (16.51)	17.33 (10.43)	31.99 (15.46)	18.22 (11.31)
Vertical RMSE (NRMSE)	99.77 (12.22)	-	73.92 (9.57)	-	181.47 (12.42)	-	351.12 (30.38)	-	179.53 (20.02)	-

**Table 5:** Bland–Altman analysis results for each gait condition. The table presents the bias, 95% confidence interval (CI) of the bias, limits of agreement (LoA), and 95% CI of the LoA for GRF and CoP estimations across the five gait conditions.

		GRF						CoP					
		LLoA, ULoA(N)		LoA 95% CI (N)		Mean Difference 95% CI (N)		LLoA, ULoA (N)		LoA 95% CI (N)		Mean Difference 95% CI (N)	
Straight	ML	−24.51	18.85	−26.68	21.02	−5.00	−0.66	−21.07	17.27	−22.98	19.18	−3.82	0.02
	AP	−24.27	25.94	−26.78	28.45	−1.68	3.34	−24.43	23.46	−26.82	25.85	−2.88	1.91
	Vertical	−172.09	249.48	−193.17	270.56	17.62	59.77						
Turn	ML	−62.05	37.33	−67.02	42.3	−17.33	−7.39	−25.72	25.33	−28.28	27.88	−2.75	2.36
	AP	−41.64	30.94	−45.27	34.56	−8.98	−1.72	−23.7	22.79	−26.02	25.12	−2.78	1.87
	Vertical	−114.07	195.23	−129.54	210.7	25.11	56.04						
Run	ML	−61.11	83.14	−68.32	90.35	3.80	18.23	−25.78	28.08	−28.47	30.77	−1.54	3.84
	AP	−816.54	81.94	−861.46	126.86	−412.22	−322.38	−21.95	25.57	−24.33	27.94	−0.57	4.18
	Vertical	−227.19	1037.9	−290.44	1101.16	342.1	468.61						
Slope (Up)	ML	−84.51	21.45	−89.81	26.75	−36.83	−26.23	−38.69	50.2	−43.13	54.64	1.31	10.2
	AP	−59.56	75.54	−66.31	82.3	1.24	14.75	−49.5	56.3	−54.79	61.59	−1.89	8.69
	Vertical	−175.02	768.67	−222.21	815.86	249.64	344.01						
Slope (Down)	ML	−35.18	39.05	−38.9	42.76	−1.78	5.64	−40.03	47.3	−44.39	51.66	−0.73	8
	AP	−66.14	50.41	−71.96	56.24	−13.69	−2.04	−42.42	45.99	−46.84	50.41	−2.63	6.21
	Vertical	−211.32	378.17	−240.8	407.65	53.95	112.9						

## Data Availability

The original contributions presented in this study are included in the article. Further inquiries can be directed to the corresponding author.

## Supplementary Materials

The following supporting information can be downloaded at: https://www.mdpi.com/article/10.3390/s25113357/s1, Table S1: Names and specifications of sensors used in load cell insertion shoes; Table S2: Computer spec; Table S3: Hyperparameters tuned in each model; Table S4: Prediction accuracy of each neural network for each gait (GRF); Table S5: Prediction accuracy of each neural network for each gait (CoP).
