# Supplementary material for: Estimation of 3D Ground Reaction Force and 2D Center of Pressure Using Deep Learning and Load Cells Across Various Gait Conditions
Source: Sensors (Basel). 2025 May 26;25(11):3357. doi: 10.3390/s25113357 (PMC12157732; doi:10.3390/s25113357)
Supplement: Supplementary file 1 [file sensors-25-03357-s001.zip › sensors-3627862-supplementary.pdf]

Supplementary Material

Estimation of 3D Ground Reaction Force and 2D Center of Pressure Using Deep Learning and Loadcells Across Various Gait Conditions

This supplementary material includes contents which are not included in the main paper due to space limit.

A. Specifications of Loadcell insertion shoes

Table S1 presents the specifications of the load cell-embedded shoes described in Section 2.1 of the main manuscript.

Table S1. Names and specifications of sensors used in load cell insertion shoes. This table provides detailed information on the types, models, and technical specifications of the sensors used in the custom shoe design.

Units	Specifications and characteristics
Loadcell	i2A Systems, Loadcell, EzForce-1D, 5V, 150kgf, 30mm x 6.9mm
Load cell data BLE transmitter	Arduino NANO, WiFi / BLE 4.2, 48MHz clock, 5V, 18mm × 45mm
Load cell data BLE receiver	Arduino NANO, WiFi / BLE 4.2, 48MHz clock, 5V, 18mm × 45mm

B. Computer spec

Table S2 is the computer specifications used to train the model in section 2.4 of the main paper.

Table S2. Names and specifications of sensors used in load cell insertion shoes. This table provides detailed information on the types, models, and technical specifications of the sensors used in the custom shoe design.

**Operating system**	**System Manufacturer**	**System Model**	**BIOS**
Window 10 Enterprise	Gigabyte Technology Co., Ltd	X570 GAMING X	F3
**CPU**	**Memory**	**GPU**	
AMD Rygen 7 3700X 8-Core Processor	32,716MB RAM	Model	Total Memory
		NVIDA GeForce RTX 2080	24,360 MB

C. Hyperparameters

Table S3 summarizes the hyperparameter types configured for the four deep learning models introduced in Section 2.4. Model performance was assessed for each setting, and the best-performing values were chosen as the final configuration.

Table S3. Hyperparameters tuned in each model

	Parameter	Value
**FNCC**	Input size	100
	n of Layers	[0, 1, 2, 3]
	n of nodes in the 1st Layer	[8, 16, 32, 64]
	Learning Rate	[0.1, 0.01, 0.001, 0.0001]
	Output size	100
	max Epochs	500(GRF), 1500(COP)
	Weight Optimization Function	Adam
	Batch Size	32
	Batch Normalization	Not used
	Dropout	Not used
**CNN**	Input size	100
	n of Hidden Layers	[1, 2, 3, 4]
	n of 1st filter size	[4, 8, 16, 32]
	Learning Rate	[0.1, 0.01, 0.001, 0.0001]
	Output size	100
	max Epochs	500(GRF), 1500(COP)
	Weight Optimization Function	Adam
	Batch Size	32
	Batch Normalization	used
	Dropout	used (0.2)
**Seq2Seq LSTM**	Input size	100
	n of Hidden Layers	[1, 2, 3, 4]
	n of 1st LSTM cell	[50,100,200,400]
	Learning Rate	[0.1, 0.01, 0.001, 0.0001]
	Output size	100
	max Epochs	500(GRF), 1500(COP)
	Weight Optimization Function	Adam
	Batch Size	32
	Batch Normalization	used
	Dropout	used (0.2)
**Transformer**	Input size	100
	n of Hidden Layers	[1, 2, 3, 4]
	n of 1st Header	[1, 2, 3, 4]
	Learning Rate	[0.1, 0.01, 0.001, 0.0001]
	Output size	100
	max Epochs	500(GRF), 1500(COP)
	Weight Optimization Function	Adam
	Batch Size	32
	Batch Normalization	used
	Dropout	used (0.2)

D. **Each model performance evaluation**

Table S4 and S5 is the computer specifications used to train the model in section 2.4 of the main paper.

Table S4 Prediction accuracy of each neural network for each gait (GRF)

		FCNN	CNN	Seq2Seq	Transformer
Straight	**layers**	4	2	**4**	2
	**units**	64	16	**100**	4
	**LR**	0.1	0.01	**0.01**	0.0001
	**ML corr**	0.57	0.90	**0.88**	0.92
	**AP corr**	0.75	0.99	**0.99**	0.99
	**Vertical corr**	0.97	0.99	**0.99**	0.99
	**ML RMSE**	51.65	13.43	**11.23**	10.08
	**AP RMSE**	51.49	13.23	**11.55**	12.94
	**Vertical RMSE**	212.67	176.65	**99.77**	100.29
Turn	**layers**	3	**2**	4	3
	**units**	64	**4**	50	16
	**LR**	0.0001	**0.001**	0.01	0.01
	**ML corr**	0.07	**0.80**	0.80	0.83
	**AP corr**	0.83	**0.98**	0.98	0.98
	**Vertical corr**	0.97	**0.98**	0.98	0.98
	**ML RMSE**	49.35	**24.32**	22.7	22.39
	**AP RMSE**	46.76	**18.1**	19.35	17.78
	**Vertical RMSE**	263.1	**73.92**	95.98	94.44
Run	**layers**	3	2	**4**	4
	**units**	64	8	**100**	16
	**LR**	0.1	0.0001	**0.01**	0.001
	**ML corr**	0.13	0.89	**0.91**	0.9
	**AP corr**	0.31	0.93	**0.93**	0.94
	**Vertical corr**	0.92	0.94	**0.94**	0.94
	**ML RMSE**	101.91	34.27	**35.48**	36.27
	**AP RMSE**	165.33	33.28	**36.44**	32.31
	**Vertical RMSE**	465.37	327.49	**351.12**	358.07
Slope (up)	**layers**	3	2	**4**	4
	**units**	64	8	**100**	16
	**LR**	0.1	0.0001	**0.01**	0.001
	**ML corr**	0.13	0.89	**0.91**	0.9
	**AP corr**	0.31	0.93	**0.93**	0.94
	**Vertical corr**	0.92	0.94	**0.94**	0.94
	**ML RMSE**	101.91	34.27	**35.48**	36.27
	**AP RMSE**	165.33	33.28	**36.44**	32.31
	**Vertical RMSE**	465.37	327.49	**351.12**	358.07
Slope (down)	**layers**	3	**4**	4	4
	**units**	32	**32**	50	4
	**LR**	0.01	**0.001**	0.01	0.01
	**ML corr**	0.53	**0.76**	0.74	0.72
	**AP corr**	0.31	**0.89**	0.89	0.91
	**Vertical corr**	0.9	**0.91**	0.91	0.91
	**ML RMSE**	176.14	**17.12**	13.23	13.06
	**AP RMSE**	99.78	**31.99**	28.27	26.5
	**Vertical RMSE**	214.04	**179.53**	242.87	180.35

Table S5 Prediction accuracy of each neural network for each gait (CoP)

		FCNN	CNN	Seq2Seq	Transformer
Straight	**layers**	2.00	2.00	**4.00**	4.00
	**units**	8.00	4.00	**200.00**	8.00
	**LR**	0.001	0.001	**0.001**	0.001
	**ML corr**	0.76	0.89	**0.86**	0.87
	**AP corr**	0.99	0.99	**0.99**	0.99
	**ML RMSE**	0.87	0.94	**0.93**	0.93
	**AP RMSE**	20.80	6.00	**5.64**	4.76
Turn	**layers**	35.53	10.89	**10.12**	13.38
	**units**	28.17	8.45	**7.88**	9.07
	**LR**	3	1	**3**	2
	**ML corr**	16	4	**100**	2
	**AP corr**	0.1	0.0001	**0.01**	0.01
	**ML RMSE**	-0.20	0.65	**0.65**	0.65
	**AP RMSE**	0.93	0.99	**0.99**	0.99
Run	**layers**	0.36	0.82	**0.82**	0.82
	**units**	19.39	6.84	**7.16**	7.11
	**LR**	21.81	9.41	**8.48**	8.58
	**ML corr**	20.60	8.12	**7.82**	7.84
	**AP corr**	4	4	**2**	3
	**ML RMSE**	64	32	**200**	16
	**AP RMSE**	0.0001	0.1	**0.01**	0.001
Slope (up)	**layers**	0.10	0.49	**0.51**	0.48
	**units**	0.77	0.77	**0.79**	0.79
	**LR**	0.43	0.63	**0.65**	0.64
	**ML corr**	10.31	7.01	**6.27**	6.41
	**AP corr**	14.36	10.43	**10.04**	9.97
	**ML RMSE**	12.33	8.72	**8.15**	8.19
	**AP RMSE**	4.00	3.00	**1.00**	4.00
Slope (down)	**layers**	16.00	32.00	**100.00**	16.00
	**units**	0.1	0.0001	**0.01**	0.01
	**LR**	-0.34	0.16	**0.22**	-0.08
	**ML corr**	0.93	0.93	**0.93**	0.93
	**AP corr**	0.30	0.55	**0.57**	0.42
	**ML RMSE**	15.13	10.98	**10.52**	10.29
	**AP RMSE**	27.09	15.74	**17.33**	16.00
